# Pulmonary Fibrosis after Pegylated Liposomal Doxorubicin in Elderly Patient with Cutaneous Angiosarcoma

**DOI:** 10.1155/2016/8034832

**Published:** 2016-01-20

**Authors:** Marco Mazzotta, Raffaele Giusti, Daniela Iacono, Salvatore Lauro, Paolo Marchetti

**Affiliations:** Medical Oncology Unit, Sant'Andrea Hospital, Via di Grottarossa 1035-39, 00189 Rome, Italy

## Abstract

*Introduction*. Angiosarcoma is a rare cancer of the inner lining of blood vessels and can arise anywhere in the body, most commonly presenting as cutaneous disease in elderly patient, involving head and neck (H&N), especially the scalp. Pegylated liposomal doxorubicin (PLD) is one of the available treatments in patients with advanced or metastatic disease. Common toxicities are myelosuppression, palmar-plantar erythrodysesthesia, nausea, and stomatitis. Regarding PLD-related pulmonary fibrosis in an uncommon toxicity, there are few cases reported in literature. None of these occurred in angiosarcoma.* Methods*. This is a case report describing an elderly patient treated with PLD for advanced H&N cutaneous angiosarcoma who developed G5 pulmonary toxicity after the second PLD administration.* Results*. According to our data and patient clinical outcome, we believe that she passed away from fatal PLD-induced pulmonary fibrosis. This is the first case of fatal interstitial pneumonitis in a 77-year-old woman treated with PLD for angiosarcoma. The case has been reported for its rarity.* Conclusions*. Pathophysiology of this phenomenon is still unclear and more studies are necessary to understand the true incidence of pulmonary toxicities in patients in treatments with PLD and its mechanism.

## 1. Introduction

Angiosarcoma is an aggressive subtype of soft tissue sarcoma, originating from endothelial-cell of vascular or lymphatic vessels. We know that the optimum treatment has not been defined. Generally, radical surgery and postoperative radiotherapy are advocated to treat patients with these tumors. In many patients, surgery often is not feasible because of the multifocal nature and local spread pattern of these tumors and chemotherapy is the primary treatment option for metastatic angiosarcoma, but the evidences are limited. The main drug groups used are anthracyclines, taxanes, and alkylating agents [1, 2].

Pegylated liposomal doxorubicin (PLD) is a liposome-encapsulated form of the doxorubicin and, as other cytotoxic antibiotics, induces the inhibition of the synthesis of DNA, RNA, and proteins. Liposomal delivery of doxorubicin improves drug penetration into tumors and decreases the drug clearance, thereby increasing the duration of the therapeutic effect. Pegylation protects the liposomes from detection by the mononuclear phagocyte system 3 and provides a stabilization effect that reduces adhesion to cells, blood vessel walls, and other surfaces. Pegylated liposomal anthracyclines were developed to decrease the risk of cardiotoxicity experienced with conventional doxorubicin while preserving the antitumor efficacy. The most common toxicities of PLD are myelosuppression (65%), palmar-plantar erythrodysesthesia (51%), nausea (46%), and stomatitis (41%) [3].

We present a case of 77-year-old Caucasian woman who developed fatal pulmonary fibrosis during treatment with pegylated liposomal doxorubicin (PLD) for advanced cutaneous angiosarcoma.

Though this toxicity from PLD has been reported in other tumors [4–7], to our knowledge, our experience may be the first case reported about this rare pattern of toxicity from PDL in a patient with soft tissue sarcoma.

## 2. Case Presentation 

**Figure 1:**
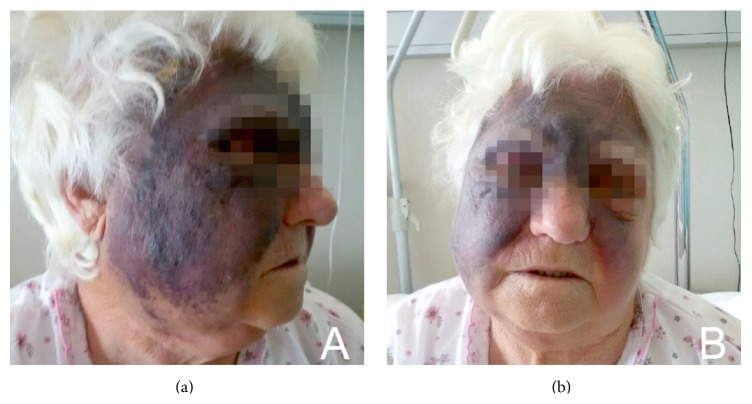
Lateral (a) and frontal (b) views show skin hyperpigmentation of the right temporal region with edematous appearance.

A 77-year-old never smoker woman presented with skin hyperpigmentation in the facial right temporal region and acute pain in the same area (Figures 1(a) and 1(b)). She also reported early breast cancer diagnosed 5 years before and treated with surgery, radiation therapy, and adjuvant Anastrozole. Hypertension, dendritic-herpes-correlated keratitis on treatment with acyclovir, and mild grade systemic sclerosis presenting with calcinosis and Raynaud's phenomenon, with no other systemic signs and no under medical treatments, were her main comorbidities.

**Figure 2:**
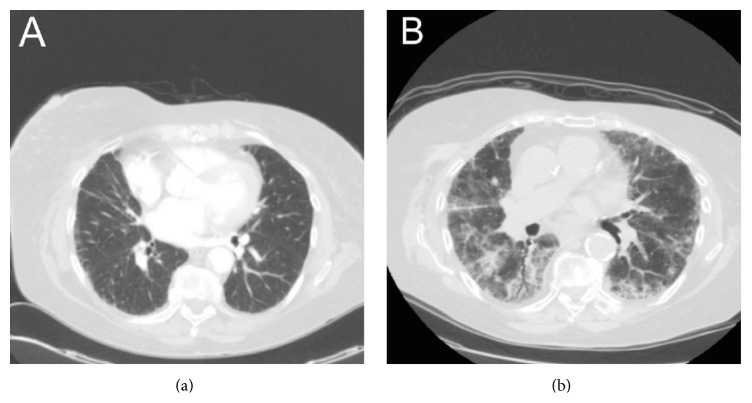
Chest CT scan at baseline (a) and after second course of chemotherapy administration, with the replacement of lung tissue by connective tissue (b).

Skin lesion biopsy suggested diagnosis of cutaneous angiosarcoma; magnetic resonance imaging (MRI) of the head and body computed tomography (CT) scan was performed to evaluate the extension of angiosarcoma: no distant metastasis and no evidence of areas of consolidation or parenchymal lesion in thoracic fields were evident (Figure 2(a)).

According to literature we decided to start chemotherapy with intravenous (iv) PLD 50 mg per square meter on day 1 every 28 days. No dose limiting toxicities were reported after the first administration [2, 8, 9].

Prophylaxis with granulocyte colony-stimulating factor (G-CSF) was not performed.

On day 5 after the 1st chemotherapy the patient presented with high temperature (38.5–39.0°C) and she was admitted again to our oncology unit for supportive care. A blood culture was performed and* Enterococcus faecalis *bacteraemia was observed. So we started iv antibiotic therapy with amoxicillin/clavulanic acid and, after complete clinical remission, second cycle of chemotherapy was administered.

Patient was readmitted to our unit on day 24 after 2nd chemotherapy because of fever, dyspnea, dry cough, shortness of breath, asthenia, and diarrhea.

Karnofsky Performance Status (KPS) estimated was 70%. Physical examination showed bilateral crackles at the bases of both lungs. Oxygen saturation was at 85% on room air and arterial blood gas showed hypoxia with pO_2_ 65 mmHg (pCO_2_ 32 mmHg pH 7.37).

According to a pneumology consult, oxygen therapy was started at 8 L/min, and the oxygen saturation improved at 96%. Blood test showed leukocytosis and high inflammation markers (erythrocyte sedimentation rate 75 mm/h, C-reactive protein 20.78 mg/dL, and procalcitonin 2.21 ng/mL). On day 26, blood culture was negative; thus we started empiric-antibiotic therapy (penicillin/tazobactam, glycopeptides, and fluoroquinolones) and high-dose intravenous corticosteroid therapy consisting of iv dexamethasone 16 mg/die; body CT scan showed altered parenchymal density in both lung fields, characterized by diffuse and confluent ground glass areas, related to an interstitial inflammatory nature; no signs of pulmonary embolism were found. An echocardiogram was performed in order to exclude heart failure and it showed no signs of cardiac distress, with an ejection fraction measurement of about 61%.

Despite the clinical situation, on day 29 scheduled head MRI showed a significant reduction of the extension of skin angiosarcoma. A chest X-ray performed after five days from the beginning of the clinical deterioration showed the worsening of pulmonary condition. On day 30 we decided to repeat chest high-resolution CT scan showing worsening of lung interstitial implication with marked thickening (Figure 2(b)).

A second line of antibiotic therapy was performed (carbapenem and macrolides) and steroid therapy was increased (iv dexamethasone 20 mg/die). Intravenous antifungal therapy was started too.

On the 10th day of hospitalization (corresponding to day 34 after 2nd chemotherapy) oxygen saturation decreased at 83% on room air. Arterial blood gas showed severe hypoxia (pO_2_ 49 mmHg, paCO_2_ 29 mmHg, pH 7.37, base excess −7.4, and HCO_3_^−^ 16) and, according to anesthesiological consult, we decided to perform noninvasive ventilation (NIV) therapy, with no improvement of respiratory failure.

A lung biopsy was not performed because of the quickly progressive worsening of the patient's general conditions and there was no indication for admission in intensive care unit by anesthesiology, according to clinical situation, age, and prognosis of the patient.

The patients died for respiratory failure after 24 days from the beginning of the symptoms.

According to our data and patient clinical outcome, we believe that she passed away from fatal PLD-induced pulmonary fibrosis.

## 3. Discussion 

Pegylated liposomal doxorubicin (PLD) is a liposome-encapsulated form of the hydrochloride salt of the anthracyclines antineoplastic antibiotic doxorubicin. Liposomal coat of phospholipids evades detection and destruction by the immune system. Pegylated form is useful to remain longer in the blood circulation. As doxorubicin is famous for its cumulative cardiac toxicities, PLD does not have this feature: its toxicity profile is remarkable for myelosuppression (65%), palmar-plantar erythrodysesthesia (51%), nausea (46%), and stomatitis (41%) [3].

Interstitial lung disease (ILD) is not listed as the toxicities related to PLD infusion and only few cases of pulmonary fibrosis after PLD chemotherapy are reported in literature (Table 1) [4–7].

Drug-induced lung injury may involve the airways, lung parenchyma, mediastinum, pleura, pulmonary vasculature, and/or the neuromuscular system. The most common form of drug-induced lung toxicity is drug-induced interstitial lung disease (DILD).

Patients likely to develop DILD are those receiving chemotherapy, those with inflammatory conditions such as rheumatoid arthritis and inflammatory bowel disease, and those receiving concurrent multiple toxic agents. Some of the known risk factors are as follows: extremes of age (i.e., childhood and old age), female sex, smoking history, drug cumulative dose, drug interaction, previous irradiation, and underlying lung disease [10].

Other cancer drugs can lead to pulmonary fibrosis, like bleomycin, taxanes, and biological agents like epidermal growth factor receptor (EGFR) small-molecule tyrosine kinase inhibitors (TKIs), monoclonal antibodies to VEGF, and mammalian target of rapamycin (mTOR) inhibitors [11]. An immune-mediated reaction, amplification of inflammatory cytokines, oxidative damage, and genetic susceptibility are likely implicated in the development of this event, but the real mechanism remains unclear.

Pulmonary fibrosis is a chronic progressive disease of lower respiratory tract. It can be diagnosed by clinical (cough, shortness of breath, hypoxia, and fatigue) and radiological signs (reticular opacities, honeycombing, and bronchiectasis), but only biopsy can confirm the diagnosis; but it is incurable, with an unstoppable slow development.

As the name implies, the histological abnormalities that characterize DILD generally involve the pulmonary interstitium to a greater extent than the alveolar spaces or airways. Generally when the interstitium acts to any drug injury, the lung must respond to the damage and repair itself. If the exposure persists or if the repair process is imperfect, the lung may be permanently damaged, with increased interstitial tissue replacing the normal capillaries, alveoli, and intact interstitium. Histological changes for most drug reactions are nonspecific.

Drugs can produce virtually all histopathological patterns of interstitial pneumonia, including hypersensitivity pneumonitis (HP), organizing pneumonia (OP), diffuse alveolar damage (DAD) and nonspecific interstitial pneumonia (NSIP), eosinophilic pneumonia, bronchiolitis obliterans organizing pneumonia (BOOP), pulmonary hemorrhage, and granulomatous pneumonitis [10].

Otherwise, pulmonary fibrosis is reported in some rheumatology diseases, including systemic sclerosis (reported in the medical history of the patient). But in our case, the acute clinical deterioration (few days after started systemic chemotherapy) and the absence of signs of pulmonary fibrosis at baseline make our diagnosis stronger.

Furthermore, the patients did not have other symptoms or signs that usually appear in final stage of sclerosis systemic (dysphagia and renal and cardiac failure) and steroid treatment did not improve the patient's clinical condition.

At last, laboratory tests carried out for the detection of anti-scl-70 and anti-nuclear antibodies definitively excluded the diagnosis of systemic sclerosis.

Also infections can have a role in development of pulmonary fibrosis, but in our case the clinical situation did not improve with antibiotic and steroid therapy and in the last hospitalization the blood culture was negative.

The patient's medical history reported thoracic radiation therapy for early breast cancer, preformed 5 years prior to the diagnosis of angiosarcoma, and this can also be a risk factor that can lead to the development of interstitial fibrosis.

The timing of this case suggests the primary role of PLD chemotherapy in the final respiratory failure by interstitial pulmonary fibrosis.

However, the absence of a histological diagnosis of DILD represents a limitation of this case report.

At this time, our experience may be the first case described about fatal interstitial pneumonitis in a 77-year-old woman treated with PLD for cutaneous angiosarcoma.

More studies are necessary to understand the true incidence of pulmonary toxicities in patients in treatments with PLD and its mechanism. Probably the pegylation and liposomal coat of the drug can help the development of a lung injury in microvessels and interstitial tissues. Furthermore the cytotoxic action of PLD can induce an immune-mediated reaction in the lung that, in patients with an appropriate medical history, can improve the establishment of this mechanism.

Oncologists should always think about development of interstitial fibrosis at first clinical signs of respiratory failure in patients under PLD treatment. Although in an oncological patient under chemotherapy with a respiratory failure infectious pneumonitis is the first suspect, we should investigate the interstitial fibrosis in order to early diagnose it and to prevent its fatal progression. Many reports in literature describe the establishment of the first symptoms of interstitial fibrosis after the second or third cycle of chemotherapy [4–6], but a slower clinical development of this condition with other agents (i.e., TKIs) is known. The radiologic examinations can be negative at the beginning: the clinical observation (cough, dyspnea, oxygen saturation, physical examination, and infections) and the medical history of patients (radiation therapy, immune-related diseases) should lead to an early clinical diagnosis that should be confirmed by CT scan (honeycombing or ground glass areas, traction bronchiectasis, and basal lesions with peripheral distribution) or by a lung biopsy.

## Figures and Tables

**Table 1:** Comparison between the present case report and the others reported in the literature.

	Age	Sex	Risk factors	Tumor	Cancer treatment	Dose of cancer treatment	Timing of symptoms development	CT scan evidence	Treatment of ILD	Outcome
Mazzotta et al.	77 y	F	Systemic sclerosis; thoracic radiation therapy	Angiosarcoma	PLD	PLD: 40 mg/m^2^ d1 q28	8 weeks after the first administration	Confluent bilateral ground glass areas	Steroids and antibiotics	Fatal

Huober et al. [4]	53 y	F	None	Breast cancer	PLD + Bevacizumab	PLD: 20 mg/m^2^ d1 q14; Bevacizumab: 10 mg/m^2^ d1 q14	6 weeks after the first administration	Ground glass areas	Steroids	Resolved

Mark and Thürlimann [5]	70 y	F	Previous chemotherapy; thoracic radiation therapy	Breast cancer	PLD	PLD: 20 mg/m^2^ d1 q14	6 weeks after the first administration	Bilaterally pronounced alveolitic changes	Steroids and antibiotics	Fatal

Inaba et al. [6]	48 y	F	Previous chemotherapy	Ovarian cancer	PLD	PLD: 50 mg/m^2^ d1 q28	8 weeks after the first administration	Ground glass areas	Steroids and antibiotics	Resolved

Nevadunsky et al. [7]	57 y	F	Previous chemotherapy	Uterine papillary serous carcinoma	PLD	PLD: 40 mg/m^2^ d1 q28	12 weeks after the first administration	Diffuse airspace disease	Steroids and antibiotics	Fatal
